# Effects of Species-Specific Auditory Stimulation on Broiler Embryos on Hatchability, Developmental Stability, Behavior, and Performance Characteristics

**DOI:** 10.3390/ani13233739

**Published:** 2023-12-03

**Authors:** Doğan Narinç, Ali Aygun

**Affiliations:** 1 Department of Animal Sciences, Faculty of Agriculture, Akdeniz University, Antalya 07100, Turkey; 2 Department of Animal Sciences, Faculty of Agriculture, Selçuk University, Konya 42100, Turkey; aaygun@selcuk.edu.tr

**Keywords:** incubation, hatching traits, auditory stimulation, chick quality, broody hen, poultry-specific sound

## Abstract

**Simple Summary:**

In nature, chicks under a broody hen hatch within a hatching window for a total of 8–10 h. However, in artificial incubation, this period is extended up to 48 h. This situation causes significant economic losses in the production industry. All behaviors performed by broody hens are imitated in artificial incubation, but only maternal sounds are absent. In this study, species-specific auditory stimulation (broody hen sound and embryo sound) was given to broiler embryos starting from the 444th and 468th hours of incubation. As a result, the hatching window decreased, chick quality improved, and the number of discarded chicks decreased in the auditory stimulation group starting from the 444th hour of incubation.

**Abstract:**

The aim of this study is to expose broiler embryos to species-specific sounds from the 444th and 468th hours of incubation until the end of incubation and, thus, to determine the effects of these stimulations on their hatching characteristics, performance traits, developmental stability, and behavioral characteristics. Auditory stimulation sounds are a total of 5 min of recording consisting of sounds made by embryos during and after internal piping and response sounds made by the broody hen at that time. The auditory stimulation pattern was created as 5 min of recording and 5 min of silence for a total of 20 min of recording, and this sound pattern was played continuously with 65 dB sound intensity and 800 Hz sound frequency. A total of 750 Ross 308 broiler hatching eggs were equally divided into three groups (AS1: auditory stimulation from hour 444, AS2: sound stimulation from hour 468), and two stimulation groups and a control (silent) group were incubated in three homologous incubators. Due to auditory stimulation, the hatching window in embryos exposed to species-specific sounds beginning at the 444th hour of the incubation period was determined to be 28 h in this study. Auditory stimulation was late in the embryos exposed to species-specific sounds from the 468th hour of incubation, and the incubation windows in this group and the silent (control) group were determined to be 36 h and 40 h, respectively. The chicks that were exposed to early auditory stimulation during incubation exhibited a higher average Tona score (99.03) in comparison to the other groups (*p* < 0.05). Additionally, the number of chicks discarded was comparatively higher than the others (*p* < 0.05). Auditory stimulation during incubation had no effect on live weight, Gompertz growth curve parameters, feed conversion ratio, slaughter-carcass characteristics, behavioral traits, or developmental balance characteristics. Consequently, it was determined that the incubation window and the number of marketable chicks were both substantially impacted by the implementation of species-specific auditory stimulation. However, further research is required to ascertain the precise timing of this auditory stimulation.

## 1. Introduction

Due to extensive genetic improvement research spanning many years, contemporary broiler chickens possess a genetic architecture that is regarded to be excellent in feed utilization, meat quality, and growth capability. Numerous environmental factors, including feed technology, thermal environment, equipment, maintenance, and management, contribute to the phenotypic manifestation of broilers’ superior genetic structure to the maximum extent possible. During the average 42-day growing period, in conventional broiler production, the physiological and environmental requirements of the birds are known nearly every day, and every requisite measure is implemented to ensure their complete fulfillment. In recent years, some practices have been implemented with the objective of enhancing the health, welfare, and post-hatching performance of birds throughout the 21-day incubation period, which is equivalent to half of the fattening period [1]. One of the applications mentioned that the objective of in ovo nutrition techniques is to provide the embryo with essential or supplementary nutrients throughout its developmental phase. Furthermore, applications of epigenetic adaptation are carried out to endow the embryos with the ability to withstand detrimental environmental conditions subsequent to their hatching [2]. Apart from these, attempts have also been made to increase the quantity of salable and healthy chicks through research initiatives that seek to enhance hatching characteristics and chick quality. The purpose of all these studies is to provide consumers with higher-quality products and generate economic benefits for the producer. 

The duration of egg incubation in industrial incubators can exhibit significant variation [3]. In contrast to the optimal circumstance wherein all chicks hatched simultaneously, this particular state of affairs results in substantial financial setbacks [4]. Studies have been made to reduce the hatching window in an effort to circumvent this issue. In these studies conducted on incubation, the effects of temperature changes [5], CO_2_ concentration [6], and different temperature profiles during incubation periods [7] were examined. In nature, broody hens provide suitable environmental conditions for their eggs, and the chicks hatch in a short hatching window. Broody hens perform some thermal stimulations, make some noises, and produce a variety of movements in order to mitigate variations in development and incubation duration [8]. Greenlees [9] claimed that while the chick was penetrating the air cell membrane of the egg, the sounds made by the chicken delayed the process of hatching, and thus, most embryos hatched synchronously and well developed. Bird embryos produce the first sounds when piercing the inner membrane, and through the syrinx, the sounds gradually turn into a species-specific sound [10]. Thus, the hatching time is shaped by the influence of both maternal sounds and the sounds produced by the embryos during incubation. During artificial incubation, all environmental conditions are kept constant, and all maternal effects are disabled. Therefore, in industrial incubation, the hatching window can be 48 h long due to genetic and processing differences between egg batches. In addition, this spread in incubation affects the time when day-old chicks first access feed and water and, therefore, their growth [4,11,12,13,14,15,16,17,18].

The initiation of auditory system functionality in chicken species is known to occur during the tenth and twelfth days of incubation [19]. According to a study by Jones et al. [20], chicken embryos begin to react to external sound stimulation at decibel levels below 90 beginning on the sixteenth day. A recent study by Bamelis et al. [21] conducted in line with prior studies on the acoustics of chickens assessed sound responses throughout the incubation period and derived biorhythm and timing indicators for the incubation procedure. The primary objective of research pertaining to sound stimulation during the incubation phase of poultry has been to establish synchronization for the process of hatching [21,22,23]. There are few studies investigating the effects of sound stimulation during incubation on the productivity, behavior, and welfare characteristics of birds in the post-hatching period. In these studies, carried out to synchronize hatching time, there are conflicting results in terms of hatching characteristics and behavioral traits. The aim of this study is to determine the incubation characteristics, general and special behavioral traits, growth performance, developmental stability (symmetry in bilateral characteristics), and slaughter-carcass characteristics of broilers by giving two different stimulations with species-specific sounds (the sounds made by the embryos during and after internal pipping and the response sounds given by the broody hen) at a sound frequency of 800 Hz under a sound intensity of 65 dB from the 444th and 468th hours of incubation until hatching.

## 2. Materials and Methods

The experiment was conducted at the Department of Animal Science, Akdeniz University in Türkiye. The care and usage of birds complied with Turkish laws, and regulations and were approved by the Ministry of Food, Agriculture, and Livestock and Akdeniz University’s Animal Experiments Local Ethics Committee (295708). This study’s animal samples consisted of chicks obtained from 750 hatching eggs acquired concurrently from a commercially available Ross 308 breeder flock at 40 weeks of age. The eggs that were obtained were not subjected to storage. Prior to incubation, a six-hour rest was performed, followed by a preheating period of six hours at a seasonally adjusted temperature of 27 °C and 55% relative humidity. Three identical incubators (VGS, Turkey), each capable of holding 500 poultry eggs, were utilized for the incubation process. The incubators are fully automated (10 floors with temperature-adjustable heating and cooling engines, an integrated moisture source for relative humidity, air circulation, and tray-turning engines per floor). During the incubation period, temperature and humidity records were made periodically with data loggers placed inside the machines. Experimental groups in this study include the following: control (C), auditory stimulation 1 (AS1), and auditory stimulation 2 (AS2). The incubators were placed in three different rooms, independent of each other and soundproofed. In the control group, no sound stimulation was performed, and a standard quiet environment (only the internal sound of the incubator during operation was constant in all three machines) was provided from the beginning to the end of the incubation. The auditory stimulation to be applied to the embryos in the experimental groups consisted of a sound pattern as follows at a sound frequency of 800 Hz under a sound intensity of 65 dB:

a The sounds made by the embryos during and after internal pipping and the response sounds made by the broody hen at that time (5 min).

b Five min of silence. 

c The sounds made by the embryos during and after internal pipping and the response sounds made by the broody hen at that time (5 min). 

d Five min of silence. 

This sound pattern (a–d) was recorded for a total of 20 min and was applied consecutively and continuously after the start of the application time (444th hours of incubation in AS1, 468th hours of incubation in AS2) and continued until the end of the incubation. Guzack IPX7 brand waterproof and remotely connected humidifier speakers placed in the middle of the incubators were used for auditory stimulation of the embryos. On the 18th day of this study, fertility control was carried out with the help of a lamp. In the AS1 experimental group, after a standard quiet environment was provided for the first 18.5 days of incubation, auditory stimulation was applied from the 444th hour until the end of incubation. In the AS2 experimental group, after a standard quiet environment was provided for the first 19.5 days of incubation, auditory stimulation was applied from the 468th hour until the end of incubation. The sounds in the incubators were measured, and the total sound intensities and frequencies were optimized. Sonic measurements were made in the machines throughout the incubation using the Loyka Lyk Bgm1356 brand device (China).

The incubators provided a temperature of 37.5 °C and 55% relative humidity for the first 18 days and a temperature of 37.2 °C and 70% relative humidity for the last three days. In the experimental groups, the number of chicks hatched every three hours after the 444th hour of incubation was determined; thus, hatching window and time-dependent changes were determined. Incubation was completed at the end of 525 h, and embryonic deaths were determined according to their periods by macroscopic examination of the eggs that did not hatch in the experimental groups. Homogeneous environmental conditions were provided to all trial groups to eliminate or equalize factors such as feed, temperature, light, and some stressors that may have an impact on the chicks. Wing numbers were assigned to hatched and dried chicks, hatching weights were determined, and then, chick quality scores were determined by the Tona score method [24]. All hatched chicks were examined by experienced operators to determine Tona chick quality score of chicks as previously described by Tona et al. [24]. Tona chick quality method is qualitative scoring system that assesses total score index of 100, respectively, based on a wide variety of visual parameters, such as activity, appearance, retracted yolk, eye condition, leg and feet condition, navel deformities, status, remaining egg membrane, beak condition, and remaining yolk.

Thanks to the wing numbers, all measurements were recorded individually throughout this study. A total of 150 chicks randomly selected from each experimental group were reared in three replicates in floor cages with a stocking density of 12 chicks/m^2^. A starter diet with 24% crude protein and 3050 kcal of metabolizable energy/kg was employed for the first 21 days, followed by a finishing diet with 23% crude protein and 3200 kcal of metabolizable energy/kg. From hatching until the end of the trial, ad libitum feeding, water, and a 23,5-hour-per-day lighting schedule were utilized.

To obtain the estimates of individual growth curve parameters, all birds were weighed weekly from hatching to 6 weeks of age using a digital scale (±0.01 g). Birds were fasted for 4 h before weighting. Due to technical limitations, the feed intake (FI) was measured in each group (30 individuals consuming from the same feeder) and feed conversion ratio (FCR) was measured individually for all chicks. FI and FCR were determined weekly and recorded for 1, 2, 3, 4, 5, and 6 weeks. Using these data (feed intake and body weight gain), cumulative feed consumption and cumulative feed conversion ratio for each bird were calculated for ages 5 and 6 weeks.

In many previous studies, it has been reported that the Gompertz function is the most compatible growth model for broiler chickens [25]. The Gompertz non-linear regression model (1) was used to estimate growth curve of each bird.

(1) $$y_{t}=\beta_{0}e^{\left(-\beta_{1}e^{-\beta_{2}t}\right)}$$

where $y_{t}$ is the weight at age *t*, $\beta_{0}$ is the asymptotic (mature) weight parameter, $\beta_{1}$ is the scaling parameter (constant of integration), and $\beta_{2}$ is the instantaneous growth rate (per day) parameter [26]. The Gompertz model is characterized by an inflection point in a manner such that $\beta_{0}/e$ of the total growth occurs prior to it and the remainder occurs after. The coordinates of the point of inflection, age, and weight at the inflection point (IPA and IPW, respectively) were obtained as follows:

(2) $$IPA=\beta_{0}/e$$

(3) $$IPW=ln\left(\beta_{1}\right)/\beta_{2}$$

The general behavioral characteristics of birds were determined using focal sampling method on the behaviors of five male and six female chicks from each experimental group for five minutes, in the morning and evening, when the birds were four weeks old, using a digital camera. The focal sampling technique was employed to determine the time budgets for the following general behavioral traits: feeding, walking, drinking, scratching, standing, shaking, cleaning, aggressive pecking, wing stretching, lying, and jumping [27]. The camera recordings were subsequently converted into data under the supervision of three laboratory specialists positioned on a sizable monitor.

Developmental stability measurements were made to determine the general stress level of the chickens in the experimental groups. Digital calipers measuring bilateral morphological characteristics (e.g., wings, shank lengths, and shank diameters) were employed to assess these attributes when the birds were six weeks old. The calipers had a precision of 0.01 mm. The relative asymmetry values (calculated as the ratio of the difference between the left and right sides of the absolute expression and expressed as a percentage) and symmetry status of bilateral characteristics (directional, asymmetrical, fluctuating) were ascertained. In order to ascertain the type of symmetry, the statistical significance of the mean of the group’s differences from zero was determined using the one-sample T-test. In a similar vein, the Shapiro–Wilk test was employed to determine whether the differences among the groups followed a normal distribution. On the basis of the results, the symmetry type of the group was determined [28]. This methodology categorizes the form of symmetry that occurs when the distribution is normal, and the mean is zero as fluctuation asymmetry. Directional asymmetry is the type of symmetry that corresponds to a normal distribution with a mean that differs from zero. Antisymmetry is a form of symmetry characterized by a non-normal distribution of differences [2,29].

The body weights of all chicks were recorded at 6 weeks of age, eight hours subsequent to feed withdrawal, and they were slaughtered at an experimental processing facility. The birds underwent the following procedures: manually cut, bleeding, scalding (at 55 °C for two minutes), defeathering with equipment, evisceration, and removal of the abdominal fat pad (extending from the proventriculus encircling the gizzard to the cloaca). The carcasses were then chilled in an ice-water tank and drained. The breast with bone and the residual abdominal fat were measured in weight the following day, following carcass dissection, on cold carcasses utilizing an electronic digital balance with a sensitivity of 0.01 g. Dissection and slaughter were carried out by the same experienced operators. In relation to body weight at six weeks of age, the yields of cold carcass, breast, leg, wing, and total fat pads were computed [30].

To determine whether experimental groups differed with regard to characteristics of broilers, the SAS 9.3 GLM procedure was utilized to conduct analyses of variance (SAS Institute 2009). The means of treatment were separated utilizing Duncan’s multiple range test. The level of significance for comparing the means was *p* < 0.05. The Rank transformation was applied to the data, which exhibited a non-normal distribution. The experimental groups’ binomial or ordinal data on mortality and chick quality traits were analyzed statistically using a generalized linear mixed-effects model with the logit function. To identify differences between the groups, the Tukey–Kramer method was applied using the SAS 9.3 GLIMMIX procedure.

## 3. Results

### 3.1. Incubation Results

**Figure 1:**
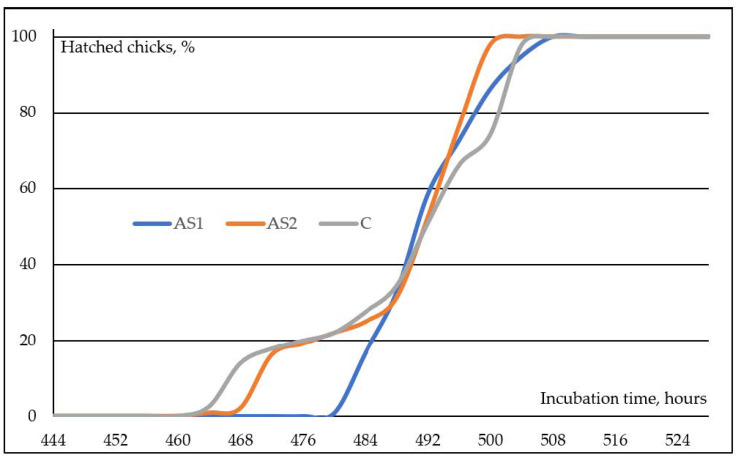
Rates of chicks hatched from eggs in trial groups depending on incubation time.

In the embryos to which auditory stimulation was applied starting from the 444th hour of incubation, the first chick hatched at the 484th hour, and the hatching time of the last chicks in the AS1 trial group was determined to be the 508th hour (Figure 1). The embryos in group AS2, which received auditory stimulation commencing starting at 468 h of incubation, exhibited the hatching of their first chick at 464 h, with the last chick hatching at 504 h, as determined by the recordings. Comparable hatching was noted in group C embryos that were not exposed to auditory stimulation; the earliest chick hatched at 464 h and the last chicks hatched at 508 h during the incubation period.

Total embryo deaths in AS1, AS2, and C groups were determined as 4.88%, 3.20%, and 3.20%, respectively (Table 1). There were no statistically significant differences between the experimental groups in terms of the average embryo mortality in either the early or late incubation periods (*p* > 0.05). Similarly, there were no statistically significant differences between the experimental groups in terms of the average hatchability of all eggs (HoT) and hatchability of fertile eggs (HoF) (*p* > 0.05).

In this study, the Tona score average value (99.03) of the chicks exposed to auditory stimulation from the 444th hour of incubation was found to be higher than the chick quality score averages of the other two experimental groups (*p* < 0.05). Similarly, second-grade chick rates were found to be higher in those exposed to auditory stimulation from the 468th hour of incubation and in the control group (*p* < 0.05). One-day-old weights of chicks obtained from AS1, AS2, and C experimental groups were 45.39 g, 44.53 g, and 45.27 g, respectively, and the chick weight averages of the experimental groups were not statistically significantly different (*p* > 0.05).

### 3.2. Performance Traits 

There was no statistically significant difference between the experimental groups in terms of live weight averages in either the fifth or sixth weeks (*p* > 0.05, Table 2). A similar situation is valid for the cumulative feed conversion ratio averages, and there were no statistically significant differences between the averages of the experimental groups in both the 5th week and the 6th week (*p* > 0.05). As a result of nonlinear regression analyses utilizing the weekly individual body weights of all birds with the Gompertz growth model, coefficients of determination (R^2^) in the range of 0.9981 to 0.9999 were found. The asymptotic weight parameter (β_0_) averages of the Gompertz growth model of the broilers in the AS1, AS2, and C experimental groups were 4957 g, 5025 g, and 5314 g, respectively, and there was no statistically significant difference between the averages (*p* > 0.05). Similarly, there were no statistically significant differences between the averages of the β_1_ and β_2_ parameters of the Gompertz model of the birds in the experimental groups (both *p* > 0.05). While the inflection point times of the Gompertz growth model of broilers in AS1, AS2, and C experimental groups were found to be 29.45 days, 29.98 days, and 30.76 days, respectively, there was no statistically significant difference between the groups (*p* > 0.05). As expected, there was no statistically significant difference between the averages of the trial groups in terms of the inflection point weight parameter, which is a function of the asymptotic weight parameter of the Gompertz growth model (*p* > 0.05).

The average values of cold carcass weight ranged between 1794 and 1813 g, and cold carcass yields ranged between 72.59 and 73.55% of the birds (Table 3), and there were no statistically significant differences between the experimental groups in terms of both characteristics (both *p* > 0.05). Similarly, the effects of auditory stimulation during incubation on the average weights of breast, leg, wing, and back were found to be statistically insignificant (all *p* > 0.05).

### 3.3. Behavioral Traits and Developmental Stability 

It was determined that the broilers in this study allocated the highest average time budget to feeding behavior (41.75–43.11%), but there was no statistical difference between the experimental group averages in terms of feeding behavior (*p* > 0.05, Table 4). A similar situation is valid for drinking behavior and other activity characteristics, and the effects of auditory stimulation in incubation on the performance and activity characteristics of broilers were found to be statistically insignificant (*p* > 0.05).

Similar to performance and activity characteristics, there were no statistically significant differences between the time budget usage averages of broilers exposed to auditory stimulation in incubation in terms of comfort and comfort behaviors (all *p* > 0.05, Table 5).

The tonic immobility averages of the broilers varied between 120.56 and 131.56 s, and there was no statistically significant difference between the experimental groups in terms of tonic immobility mean values (Table 6). There were no statistically significant differences between the experimental groups in terms of the relative asymmetry averages measured in broilers for face length, wing length, shank length, and shank diameter.

While anti-symmetry was detected in the AS2 trial group for face length, anti-symmetry was determined as the symmetry type in all trial groups for wing length (Table 7). For shank length, a fluctuating asymmetry type was found in all experimental groups, and for shank diameter, a fluctuating asymmetry type was determined only in broilers in the AS1 experimental group.

## 4. Discussion

In natural life, there are 10–15 eggs incubated under a broody hen, and the chicks obtained from this hatch within a short “hatching window”, defined as the time between the first and last chicks to hatch. Broody hen turns the embryos and applies thermal stimulations to optimize their development and minimize incubation time differences [8]. In addition, broody hen makes different sounds and communicates with the embryos, especially as it approaches the end of incubation. Thus, in natural life, the hatching window is completed within 6–8 h. During artificial incubation, all environmental conditions are kept constant, and all maternal effects are disabled. Therefore, in an industrial incubation environment, the hatching window can be as long as 48 h, although it varies depending on factors such as genetic differences between breeders, the age of the breeder flock, and the storage conditions of hatching eggs. This situation affects the access time of the first chicks to water and feed [4,11,12]. Due to auditory stimulation, the hatching window in embryos exposed to species-specific sounds beginning at the 444th hour of the incubation period was determined to be 28 h in this study. In the AS2 experimental group, which was given a species-specific sound starting from the 468th hour of incubation, the first chick hatched at the 464th hour before the application. In this experimental group, the last chick hatched at the 500th hour of incubation. The hatching window duration in the AS2 trial group was 36 h. In the control group, which included broiler embryos without any sound stimulation during incubation, the first chick hatched at the 464th hour, and the last chick hatched at the 504th hour; thus, the hatching window size in this group was determined as 40 h. As can be seen from the findings, species-specific auditory stimulation delayed chick hatching at the 444th hour of incubation, and it was understood that applying this application at later hours had no effect. Greenlees [9] claimed that the sounds made by the broody hen, while pecking the membrane of the egg’s air cell with its beak, delayed internal piping; thus, most embryos hatched synchronously and well-developed. In addition to these sounds of the broody hen, it is known that the calls made by the embryos also play a role in the synchronization of incubation [31,32]. In the current study, the findings that auditory stimulation application at the 444th hour of incubation reduced the hatching window and synchronization of hatched chicks support the claims reported by Greenlees [9] and the results of other researchers [31,32]. 

Veterany et al. [33] reported that synthetic internal pipping sounds during incubation affected hatching start time, incubation window length, and total incubation time, but did not affect hatchability. In the studies conducted by Tong et al. [34] and Veterany et al. [31], it was reported that artificial sound stimulation in incubation increased embryonic mortality. In this study, as reported by Veterany et al. [33], auditory stimulation during incubation did not affect hatchability. However, contrary to reports by Tong et al. [34] and Veterany et al. [31], the effect of auditory stimulation on embryo mortality was found to be insignificant. The reason for this situation is thought to be related to the type, intensity, and pattern of sound to which the embryos are exposed. In a study conducted by Sanyal et al. [35], it was reported that high-decibel musical stimulation moderately increased the plasma noradrenaline level and positively affected the spatial orientation, learning, and memory of one-day-old chicks. On the other hand, noise at the same sound pressure level caused the plasma noradrenaline level to increase excessively and caused deterioration in spatial behavior. Balaban et al. [36] found that chicken embryos showed selective sensitivity to broody hen vocalizations before the forebrain became active and that broody hen vocalizations caused the entire brain to become active as an integrated system earlier than expected.

In the current study, chick quality and the number of first-grade chicks were found to be higher in the group (AS1) that received auditory stimulation from the 444th hour of incubation compared to the other groups. Tong et al. [34], who performed species-specific sound stimulation on broiler embryos starting from the 10th day of incubation, reported that sound stimulation delayed the time for the first chick to hatch but had no effect on chick quality, blood traits, and plasma corticosterone concentrations. It is thought that the reason for this situation is due to the timing difference between the applications. Similarly, it was determined that the embryos in the AS2 group, which received auditory stimulation in the late period of incubation, did not differ from those in the control group in terms of chick quality characteristics. Tong et al. [34] have reported that the timing and type of auditory stimulation before hatching are important. If sounds do not have an appropriate sequence, speed, frequency, and duration according to natural timing and order, this practice can have a negative impact and cause pre-hatch stress, which has the potential to disrupt embryo development and animal welfare. 

No studies have been documented in the scientific literature examining the post-hatch performance, behavior, and developmental stability characteristics of poultry embryos exposed to auditory stimulation during the incubation period. The effects of auditory stimulation in incubation on weekly live weight and feed conversion averages were found to be insignificant. A similar situation is valid for the parameters of the Gompertz growth curve model. The averages determined in the study in terms of the above-mentioned characteristics were found to be compatible with the reports of many researchers [37,38,39,40]. Time budget averages regarding the general behavioral characteristics, the averages of tonic immobility duration, and relative asymmetry averages in the current study are consistent with previous reports of various researchers in broilers [41,42,43,44]. According to the developmental stability status detected in bilateral features, only one anti-symmetry was detected for the shank diameter feature in the AS1 group, while anti-symmetry was detected in three and two bilateral features in the AS2 and control groups, respectively. However, there is no evidence that this situation is caused by the species-specific sound application during incubation. As it is known, anti-symmetry occurs in cases of stress caused by chance environmental factors.

## 5. Conclusions

In this study, it was determined that species-specific auditory stimulation, starting from the 444th hour of incubation, reduced the hatching window, synchronized chick hatching, and improved chick quality. However, it was determined that the same application performed after the 468th hour of incubation was late and did not lead to any improvement in the mentioned characteristics. In chicken embryos, the ontogeny of the auditory system begins on the 10th day of embryonic development, and the auditory system becomes functional after the 16th day of incubation. In this case, further studies are needed to determine the most appropriate time for species-specific auditory stimulation after the 16th day of incubation.

## Figures and Tables

**Table 1:** The results of statistical analysis of the mean values of the hatching characteristics observed in the experimental groups.

Group	EEM (%)	LEM (%)	HoT (%)	HoF (%)	Tona Score	Second Grade Chicks (%)	Chick Weight (g)
AS1	1.66	3.32	93.20	96.68	99.03 ^a^	1.72 ^b^	45.39
AS2	1.20	2.00	96.00	97.96	98.75 ^b^	3.33 ^a^	44.53
C	1.20	2.00	94.40	97.93	98.77 ^b^	3.39 ^a^	45.27
SEM	0.42	0.57	0.83	0.58	0.05	0.62	0.16
*p* value	0.879	0.551	0.386	0.589	0.032 *	0.043 *	0.059

EEM: early embryonic mortality, LEM: late embryonic mortality, HoT: hatchability of total eggs, HoF: hatchability of fertile eggs, SEM: standard error of means, * *p* < 0.05, ^a,b^: Differently lettered averages within the same column differ statistically (*p* < 0.05).

**Table 2:** The results of statistical analysis of the mean values of the growth and feed efficiency characteristics observed in the experimental groups.

Group	BW5	BW6	FCR5	FCR6	β_0_	β_1_	β_2_	IPT	IPW
AS1	2250	2837	1.69	1.83	4957	4.93	0.056	29.45	1843
AS2	2235	2847	1.63	1.78	5025	4.83	0.055	29.98	1876
C	2302	2951	1.64	1.77	5314	4.83	0.053	30.76	1985
SEM	16.50	21.81	0.01	0.01	74.98	0.04	0.001	0.31	33.92
*p*-value	0.229	0.071	0.061	0.072	0.133	0.436	0.389	0.245	0.220

BW5–6: Body weights at 5 and 6 weeks of age, FCR5–6: Cumulative feed conversion ratio at 5 and 6 weeks of age, β_0_ = Asymptotic body weight parameter; β_1_ = Shape parameter; β_2_ = Instantaneous growth rate parameter, IPT = Time (day) at inflection point of growth curve, IPW = Body weight (g) at inflection point of growth curve, SEM: standard error of means.

**Table 3:** The results of statistical analysis of the mean values of the slaughter-carcass traits observed in the experimental groups.

Group	Cold Carcass (g)	Cold Carcass (%)	Breast (g)	Leg (g)	Wing (g)	Back (g)
AS1	1794	72.59	899.6	704.4	244.0	585.8
AS2	1807	73.13	905.1	706.1	241.1	595.3
C	1813	73.35	905.8	703.4	238.2	594.5
SEM	3.07	0.12	5.96	1.64	1.68	2.96
*p* value	0.691	0.687	0.945	0.795	0.377	0.337

SEM: standard error of means.

**Table 4:** The effect of auditory stimulation on performance and activity behavioral characteristics (%).

Group	Feeding	Drinking	Walking	Lying	Standing	Jumping
AS1	43.11	4.79	4.75	26.89	10.25	0.18
AS2	41.75	5.89	4.85	28.46	7.49	0.54
C	42.43	5.12	3.21	30.74	8.75	0.41
SEM	4.47	1.22	1.18	4.32	1.15	0.06
*p* value	0.547	0.689	0.554	0.798	0.456	0.122

SEM: standard error of means.

**Table 5:** The effect of auditory stimulation on comfort and social behavioral characteristics (%).

Group	Cleaning	Wing Stretching	Shaking	Aggressive Pecking	Scratching
AS1	3.21	1.17	1.32	0.94	3.45
AS2	2.75	1.49	1.17	0.86	4.79
C	2.56	1.23	1.26	1.08	3.21
SEM	0.68	0.12	0.09	0.05	0.18
*p* value	0.356	0.455	0.298	0.754	0.158

SEM: standard error of means.

**Table 6:** The effect of auditory stimulation on tonic immobility (sec) and relative asymmetry (%).

Group	Tonic Immobility, Sec	Relative Asymmetry, %			
		Face Length	Wing Length	Shank Length	Shank Diameter
AS1	131.56	2.349	1.826	1.900	2.161
AS2	123.78	2.213	1.980	1.763	2.418
C	120.56	2.472	1.926	1.601	2.429
SEM	2.41	0.091	0.074	0.094	0.131
*p* value	0.162	0.517	0.677	0.441	0.634

SEM: standard error of means.

**Table 7:** The effect of auditory stimulation on developmental stability.

Group	Criteria	Face Length	Wing Length	Shank Length	Shank Diameter
AS1	Shapiro–Wilk	0.569	0.000	0.113	0.256
	One sample T	0.449	0.023	0.066	0.245
	Status	Fluctuation Asymmetry	Anti- symmetry	Fluctuation Asymmetry	Fluctuation Asymmetry
AS2	Shapiro–Wilk	0.026	0.046	0.685	0.010
	One sample T	0.400	0.003	0.071	0.215
	Status	Anti- symmetry	Anti- symmetry	Fluctuation Asymmetry	Anti- symmetry
C	Shapiro–Wilk	0.059	0.005	0.384	0.000
	One sample T	0.502	0.001	0.355	0.072
	Status	Fluctuation Asymmetry	Anti- symmetry	Fluctuation Asymmetry	Anti- symmetry

## Data Availability

The data presented in this study are available upon request from the corresponding authors.
